# Extended Ganglion Cell Layer Thickness Deviation Maps With OCT in Glaucoma Diagnosis

**DOI:** 10.3389/fmed.2021.684676

**Published:** 2021-06-04

**Authors:** Paul Lehmann, Bettina Hohberger, Robert Lämmer, Christian Mardin

**Affiliations:** Department of Ophthalmology, University of Erlangen-Nürnberg, Friedrich-Alexander-University of Erlangen-Nürnberg, Erlangen, Germany

**Keywords:** retinal ganlion cell, OCT, spectralis, glaucoma, GRID

## Abstract

**Purpose:** The aim of the present study was to investigate the diagnostic power of RGCL in the macula quantitatively and qualitatively by using a conventional and extended elliptic grid with deviation maps.

**Subjects and Methods:** Thickness of RGCL was measured using SPECTRALIS® OCT (Heidelberg Engineering, Heidelberg, Germany) in 150 eyes of 150 subjects of the Erlangen Glaucoma Registry (EGR; NTC00494923): 26 ocular hypertension (OHT), 39 pre-perimetric open-angle glaucoma (pre-OAG), 19 normal tension glaucoma (NTG), 34 primary open-angle glaucoma (POAG), 16 secondary open-angle glaucoma (SOAG), and 16 controls. Analysis of RGCL was done quantitatively (global value, GV) and qualitatively (qualitative total value, QTV) by using a color-coded point score for data of the common elliptic macular grid of deviation maps. Furthermore, qualitative analysis of RGCL was done for an extended elliptic macula grid (extended qualitative total value, eQTV). Receiver operating characteristic (ROC) curves were calculated for the conventional and the enlarged macular grid for all subjects' groups.

**Results:** GV of RGCL thickness differed significantly between pre-OAG (*p* < 0.05), NTG (*p* < 0.001), POAG (*p* < 0.001), SOAG (*p* < 0.001), yet not OHT (*p* > 0.05) and controls, respectively. Quantitative ROC analysis of GV showed AUC of 0.965 (SOAG), 0.942 (POAG), 0.916 (NTG), 0.772 (pre-OAG), and 0.526 (OHT). QTV differed significantly between pre-POAG (*p* < 0.05), NTG (*p* < 0.001), POAG (*p* < 0.001), SOAG (*p* < 0.001), yet not OHT (*p* > 0.05) and controls, respectively. Qualitative ROC analysis of QTV showed AUCs of 0.908 (NTG) 0.914 (POAG), 0.930 (SOAG), 0.734 (pre-POAG), and 0.519 (OHT). Implementation of eQTV yielded even higher AUCs for NTG (0.919), POAG (0.969), and SOAG (0.973) compared to GV. Similar AUCs of eQTV and GV were observed for OHT (0.514) and pre-OAG (0.770).

**Conclusion:** The results of the present study showed that quantitative and qualitative analysis of RGCL thickness yielded similar diagnostic impacts compared to RNFL. Qualitative analysis might be a quick and easy useable tool for clinical all-day life. The present data suggest that analysis of an extended macula region might improve its diagnostic impact.

## Introduction

Glaucoma is one of the most common causes of blindness worldwide. The health burden caused by glaucoma increased in the last 25 years (1, 2). Several risk factors are involved in the multifactorial pathogenesis of this neurodegenerative disease (3) including advanced age, positive family history, severe myopia, and its main risk factor an elevated intraocular pressure (IOP) (4, 5). It is assumed that high IOP triggers retinal ganglion cell (RGC) loss (6). Loss of RGCs occurs before functional abnormalities can be seen in a patient's perimetry (7).

Optical coherence tomography (OCT) offers the ability to examine characteristics of the retina and optic nerve head of glaucoma patients in an objective and non-invasive way (8). Thereby, scan quality reaches to the level of histological images. It seems to be more precisely than perimetry for diagnosing the progression of glaucoma in earlier stages (9). Next to measurement of the peripapillary retinal nerve fiber layer (RNFL), RGC layer (RGCL) thickness can be quantified (10). The RNFL thinning decreases in speed while glaucoma disease continues to progress. In contrast, RGCL thickness continued to decrease constantly with glaucoma progression (9). Thus, analysis of RGCL thickness might offer an additional diagnostic parameter for revealing progression of glaucoma from early to advanced stages. In eyes with early pre-perimetric glaucoma average RGCL thickness and especially the inferior region of the macula RGCL were observed to be the most appropriate ones for diagnostics (7). Implementation of enlarged grids for RGCL analysis, enabling analysis of larger macula regions, lead to an enhanced diagnostic power, as temporal quadrants of larger macular grids reached highest AUC value (11).

Most of the recent studies analyzed the ganglion cell complex (GCC), consisting of the inner plexiform layer (IPL), ganglion cell layer (GCL), and retinal nerve fiber layer (RNFL) (12) or GCIPL (i.e., GCL and IPL) (13, 14). To best of our knowledge, we did not find a study in literature focusing on single RGCL thickness in patients with different types of glaucoma. However, a finer analysis of macular retinal layers could identify distinct alterations of retinal ganglion cells. Thus, by analyzing single RGCL thickness, very fine changes in RGCL could be detected at even earlier stages of disease. The aim of the present study was to investigate the diagnostic power of only RGCL in the macula quantitatively and qualitatively by using a conventional and extended elliptic grid in patients with ocular hypertension (OHT), pre-perimetric open-angle glaucoma (pre-OAG), normal tension glaucoma (NTG), primary open-angle glaucoma (POAG), and secondary open-angle glaucoma (SOAG) compared to controls. In addition, RGCL data were compared to RNFL in patients' groups.

## Materials and Methods

### Patients

One hundred fifty eyes of 150 patients of the Erlangen Glaucoma Registry (EGR; NTC00494923) of the Department of Ophthalmology of the University of Erlangen-Nürnberg were analyzed retrospectively: 26 ocular hypertension (OHT), 39 pre-perimetric open-angle glaucoma (pre-OAG), 19 normal tension glaucoma (NTG), 34 primary open-angle glaucoma (POAG), 16 secondary open-angle glaucoma (SOAG), and 16 controls. The EGR is a longitudinal follow-up study under therapy, including subjects with manifest glaucoma, glaucoma suspects, and a control group. All patients received an ophthalmic examination, including slit lamp microscopy, fundoscopy, and gonioscopy. IOP was measured by Goldmann applanation tonometry. Visual field was tested using white-on-white Octopus perimetry (mean defect, MD; Octopus 500, 900; program G1, Interzeag, Schlieren, Schweiz, Peridata Software). Demographic data of all subjects can be seen in Table 1.

**Table 1:** Classification of perimetric glaucoma patients (NTG, POAG, SOAG) into subgroups based on mean defect (MD, Octopus perimetry): mild, moderate, and advanced; ratio represents percentage of disease severity in all perimetric glaucoma eyes.

**Severity**	**Total ratio %**	**NTG**	**POAG**	**SOAG**
Mild MD ≤6 dB	30.4	5	11	5
Moderate MD > 6 dB and ≤12 dB	33.3	10	9	4
Advanced MD > 12 dB	36.2	4	14	7

Diagnosis was done according to the following criteria:

### OHT

Diagnosis of OHT was based on an increased IOP > 21 mmHg (repeated twice). Optic nerve head and visual fields showed no pathological alterations.

### Pre-OAG

Pre-OAG showed an increased IOP > 21 mmHg (repeated twice), alterations of the optic nerve head, classified according to Jonas et al. (15) Visual field was normal.

### POAG

Diagnosis of POAG was based on an increased IOP > 21 mmHg (repeated twice), alterations of the optic nerve head, classified according to Jonas et al. Visual field defects were detected according to the following criteria: Scotomas with ≥3 neighboring test points on the pattern deviation map with a probability of <5%, ≥2 adjacent test points on the pattern deviation map with a probability of <1% and MD > 2.8 dB. These perimetric defects had to be located at the same side in at least 2 consecutive examinations.

### NTG

Diagnosis of NTG was as for POAG (see above), yet IOP was within normal ranges ≤21 mmHg.

### SOAG

Patients meeting criteria of POAG and additionally were affected by pseudoexfoliation syndrome (7) or melanin dispersion (9) were classified as SOAG.

If both eyes met the inclusion criteria, one eye of each person was selected randomly for the present analysis. Glaucoma patients were classified into 3 groups based on the severity of visual field defect in Octopus perimetry. Mild glaucoma was defined as MD ≤6 dB. Moderate glaucoma was present when the patient's MD was >6 dB and ≤12 dB. Advanced glaucoma was allocated when MD was >12 dB. Classification of patients with mild, moderate and advanced glaucoma can be seen in Table 2.

**Table 2:** Demographic data: median and quartiles [of all subgroups (OHT, pre-OAG, POAG, NTG, SOAG, controls); Gender [m/f]], Age [years], BCVA, IOP [mmHg], MD [dB], and RNFL [μm].

	**Gender [m/f]**	**Age [years]**	**BCVA**	**IOP [mmHg]**	**MD [dB]**	**Global RNFL [μm]**
OHT	13/13	60.0 (49.8–71.3)	0.8 (0.8–1.0)	17.0 (15.0–19.0)	2.18 (0.9–3.4)	94.0 (88.0–103.3)
Pre-OAG	20/19	66.0 (60.0–75.0)	1.0 (0.8–1.0)	14.0 (12.0–17.0)	2.5 (1.1–4.4)	76.0 (64.0–84.0)
NTG	6/13	75.0 (66.0–80.0)	0.7 (0.5–0.9)	10.0 (10.0–13.0)	9.1 (4.5–12.0)	62.0 (55.0–73.0)
POAG	15/19	71.0 (65.5–78.0)	0.8 (0.6–0.9)	13.0 (11.8–15.0)	9.65 (4.4–13.9)	55.5 (48.0–72.8)
SOAG	10/6	66.0 (56.0–75.3)	0.63 (0.5–1.0)	12.5 (10.0–14.8)	9.7 (5.1–17.1)	59.0 (49.3–67.3)
Controls	5/11	57.5 (51.3–66.5)	0.95 (0.8–1.0)	13.5 (12.0–16.0)	1.33 (0.2–2.7)	90.0 (87.0–96.0)

### Controls

Control eyes showed an IOP within normal ranges ≤21 mmHg, no alterations of the optic nerve head and a normal visual field. MD was ≤2.8 dB. Less than 3 adjacent test points on the pattern deviation map with a probability of <5% and <2 adjacent test points on the pattern deviation map with a probability of <1%.

**Figure 1:**
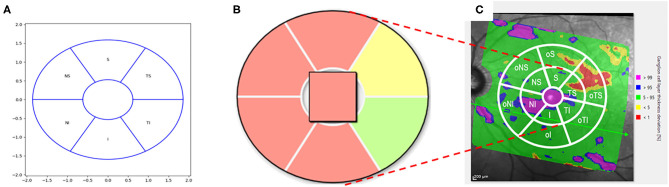
Schematic scetch of the conventional macular grid **(A)** with color coding **(B)** and extended version **(C)**. **(A)** Conventional Macular Grid: The macular grid was defined by an elliptical ring: the inner radius was 0.618 mm in the horizontal axis and the inner radius of the vertical axis was 0.531 mm. The outer radius of the elliptical ring was 1.857 mm horizontally and 1.590 mm vertically. The elliptical ring was divided into 6 sectors with angles of 60°. These 6 sectors of the macular grid corresponded to the superior (S), inferior (I), temporal-superior (TS), temporal-inferior (TI), nasal-superior (NS), and nasal-inferior (NI) macular region. **(B)** Macular Grid color-coding: The sectors of the macular grid were color-coded according to percentiles: A sector is marked with green if the thickness of the RGCL in this sector is >5th percentile of a healthy reference population. If a sector is colored yellow, the value of the average RGCL thickness < 5th percentile, yet > 1st percentile of the reference database. A red colored sector represented data which were < 1st percentile. In the qualitative analysis, a green sector was counted with one point, a yellow sector with 2 and a red sector with 3 points. (C) Extended Macular Grid: An enlarged elliptical ring was added to the pre-existing macular grid (extended macular grid): 3.714 mm horizontally and 3.18 mm vertically (i.e., doubled outer radii of the conventional macular grid, respectively). This resulted in 6 new outer sectors (o). The outer sectors were called outer superior (oS), outer inferior (oI), outer temporal-superior (oTS), outer temporal-inferior (oTI), outer nasal-superior (oNS), and outer nasal-inferior (oNI).

Thickness of the retinal ganglion cell layer (RGCL) and global retinal nerve fiber layer thickness (gRNFL) were measured using SPECTRALIS® optical coherence tomography (Heidelberg Engineering, Heidelberg, Germany). “Macular grid” was used to study the region of the macula lutea. On the basis of a 30° ×25° volume scan of the macula the macular grid is defined by an elliptical ring (Figure 1A): the inner radius was 0.618 mm in the horizontal axis and the inner radius of the vertical axis was 0.531 mm. Outer radius of the elliptical ring was 1.857 mm horizontally and 1.590 mm vertically. The elliptical ring was divided into 6 sectors with angles of 60°. These 6 sectors of the macula grid corresponded to the superior (S), inferior (I), temporal-superior (TS), temporal-inferior (TI), nasal-superior (NS), and nasal-inferior (NI) macular region. Macular grid is normalized to the axis between the center of Bruch's membrane opening of the disc and the foveola (Anatomic Positioning System, APS).

Thickness of RGCL was measured (μm) by Spectralis II (Heidelberg, Germany). Sectorial and global mean (*global value “GV”*) were presented, respectively. The sectors of the macular grid in so-called deviation maps were color-coded according to percentiles (Figure 1B): A sector is marked with green if thickness of the RGCL in this sector is >5th percentile of a healthy reference population (16). If a sector is colored yellow, the value of the average RGCL thickness < 5th percentile, yet > 1st percentile of the reference database. A red colored sector represented data which were < 1st percentile. The SPECTRALIS® retinal thickness reference database is based on data of 255 eyes of 255 healthy patients of European origin. Data in this database were corrected due for age and the distance between fovea and BMO center using a multiple linear regression model. The study was done in accordance with the Helsinki Declaration and was approved by the ethics committee of the University of Erlangen-Nürnberg.

### Extended Macular Grid

The SPECTRALIS® software measures a much larger retinal section than the central macular grid covers for RGCL analysis and these thickness deviation maps are also color-coded. Yet, only qualitative, no quantitative data of RGCL are available for this “extended” region.

An enlarged elliptical ring was added to the pre-existing macular grid (extended macular grid, Figure 1C): 3.714 mm horizontally and 3.18 mm vertically (i.e., doubled outer radii of the original macular grid, respectively). This resulted in 6 new outer sectors (o). The outer sectors were called outer superior (oS), outer inferior (oI), outer temporal-superior (oTS), outer temporal-inferior (oTI), outer nasal-superior (oNS), and outer nasal-inferior (oNI). Combing each original macular grid sector with its corresponding outer sector (for example eS = S + oS), extended sectors were created: extended superior (eS), extended inferior (eI), extended temporal-superior (eTS), extended temporal-inferior (eTI), extended nasal-superior (eNS), and extended nasal-inferior (eNI) sector.

### Statistical Analysis

Statistical analysis was done using the SPSS Version 24.0. A non-parametric test (Mann-Whitney-U-Test) was used. All results were corrected according to Bonferroni considering multiple testing. A quantitative and qualitative analysis of the RGCL thickness were done. A self-developed point score was designed for qualitative analysis (see below). To compare statistical power of different ROCs, sensitivity, and specificity were calculated. When calculating sensitivity and specificity, cut-off was selected as the point where Youden index had its maximum (i.e., optimum sensitivity and specificity) (17).

### Quantitative Analysis

Quantitative analysis measured the absolute RGCL thickness [μm] for each sector, respectively. In addition, mean of all sectors was calculated (i.e., global value; GV). Receiver Operating Characteristic (ROC) curves were performed considering each sector individually and GV.

### Qualitative Analysis

For the qualitative analysis of the RGCL a point scoring was allocated to each sector of the macula grid (Figure 1B):

- red: 3 points
- yellow: 2 points
- green: 1 point

This point scoring of all sectors was summed up (i.e., qualitative total value; QTV). ROC curves were done for each sector of the macula grid and QTV.

### Qualitative Analysis of the Extended Macular Grid

For the qualitative analysis of the RGCL thickness of the extended macular grid (Figure 1C) an additional point scoring was established. The following points were allocated for each of the 12 sectors (there had to be a cluster of at least 3 pixels to be counted valid):

- Only green (complete sector): 0 points
- Only yellow and ≤50% of the area in one sector: 1 point
- Only yellow and >50% of the area in one sector: 2 points
- Yellow and red and ≤50% of the area in one sector: 3 points
- Yellow and red and >50% of the area in one sector: 4 points
- Only red (complete sector): 5 points

The points of an extended sector were obtained by summing up the points from the outer sector with its corresponding sector from the original macular grid (for example points eS = points S + points oS). This point scoring of all extended sectors was summed up (i.e., extended qualitative total value; eQTV). ROC curves were done for each extended sector of the extended macula grid and eQTV.

## Results

### Quantitative Analysis

Median and quartiles of RGCL thickness for GV and each sector can be seen in Table 3. In addition, Table 3 shows *p*-values for all comparison groups. GV differed significantly between pre-OAG, NTG, POAG, SOAG, and controls (*p* < 0.05), respectively. Yet, no significant difference was observed between OHT and controls (*p* > 0.05).

**Table 3:** Global and sectorial RGCL thickness (median, quartiles; μm) of all subgroups (OHT, pre-OAG, NTG, POAG, SOAG, controls); GV, global value; TS, temporal superior; S, superior; NS, nasal superior; NI, nasal inferior; I, inferior; TI, temporal inferior; p-values (p) for comparison of each subgroup with controls for quantitative RGCL thickness ([μm], Bonferroni-corrected).

	**OHT**	**Pre-OAG**	**POAG**
GV TS S NS NI I TI	51.5 (47.0–55.0) *p* > 0.05 48.5 (43.6–52.0) *p* > 0.05 53.0 (48.8–56.3) *p* > 0.05 52.0 (48.0–56.0) *p* > 0.05 52.5 (48.8–56.0) *p* > 0.05 52.5 (49.0–54.0) *p* > 0.05 51.5 (47.0–53.0) *p* > 0.05	45.0 (37.0–49.0) *p* < 0.05 38.0 (29.0–47.0) *p* < 0.05 45.0 (35.0–51.0) *p* < 0.05 47.0 (41.0–52.0) *p* < 0.05 48.0 (41.0–52.0) *p* > 0.05 48.0 (40.0–51.0) *p* < 0.05 45.0 (35.0–50.0) *p* < 0.05	35.0 (27.8–40.0) *p* < 0.001 29.0 (21.0-41.0) *p* < 0.001 39.5 (30.0–45.3) *p* < 0.001 39.5 (30.8–47.5) *p* < 0.001 41.0 (32.8–44.8) *p* < 0.001 32.5 (24.8–42.3) *p* < 0.001 27.0 (19.8–40.0) *p* < 0.001
	**NTG**	**SOAG**	**Controls**
GV TS S NS NI I TI	38.0 (29.0–42.0) *p* < 0.001 31.0 (24.0–39.0) *p* < 0.001 40.0 (29.0–50.0) *p* < 0.001 43.0 (35.0–52.0) *p* < 0.001 45.0 (31.0–50.0) *p* < 0.01 34.0 (24.0–45.0) *p* < 0.001 24.0 (18.0–40.0) *p* < 0.001	29.0 (24.3–38.3) *p* < 0.001 23.5 (20.3–31.8) *p* < 0.001 29.0 (25.3–37.3) *p* < 0.001 35.5 (26.3–44.0) *p* < 0.001 33.0 (23.0–44.8) *p* < 0.001 28.5 (24.0–38.8) *p* < 0.001 23.5 (18.8–34.3) *p* < 0.001	52.0 (48.3–53.0) 47.5 (44.0–50.0) 53.0 (48.8–54.8) 53.5 (48.5–55.0) 53.0 (49.3–55.8) 52.0 (51.0–54.8) 51.0 (47.5–52.8)

ROC analysis of quantitative RGCL thickness is shown in Table 4. GV yielded highest AUCs for SOAG, POAG, and NTG vs. controls, respectively (>0.9). Sectorial analysis for these subgroups ranged between 0.882 and 0.977. AUCs of OHT vs. controls showed values between 0.5 and 0.6.

**Table 4:** AUC of ROC analysis of quantitative RGCL thickness for each subgroup (OHT, pre-OAG, NTG, POAG, SOAG vs. controls, respectively): global (GV, global value) and sectorial (TS, temporal superior; S, superior; NS, nasal superior; NI, nasal inferior; I, inferior; TI, temporal inferior).

		**TS**	**S**	**NS**	**NI**	**I**	**TI**	**GV**
OHT-controls	AUC	0.561	0.525	0.534	0.513	0.523	0.512	0.526
Pre-OAG-controls	AUC	0.748	0.763	0.742	0.708	0.78	0.795	0.772
NTG-controls	AUC	0.88	0.9	0.859	0.845	0.883	0.882	0.916
POAG-controls	AUC	0.903	0.926	0.886	0.884	0.937	0.945	0.942
SOAG-controls	AUC	0.977	0.959	0.936	0.918	0.965	0.947	0.965

### Qualitative Analysis

Median and quartiles of RGCL thickness score can be found in Table 5. *P*-values for all comparison groups are shown in Table 5. QTV of RGCL thickness score differed significantly between pre-OAG, NTG, POAG, SOAG, and controls (*p* < 0.05), respectively. Yet, not significant difference was observed between OHT and controls (*p* > 0.05).

**Table 5:** Total and sectorial RGCL thickness score (median, quartiles) of all subgroups (OHT, pre-OAG, NTG, POAG, SOAG, controls): QTV, qualitative total value; TS, temporal superior; S, superior; NS, nasal superior; NI, nasal inferior; I, inferior; TI, temporal inferior; p-values (p) for comparison of each subgroup with controls (Bonferroni-corrected).

	**OHT**	**Pre-OAG**	**POAG**
QTV TS S NS NI I TI	0.0 (0.0–0.0) *p* > 0.05 0.0 (0.0–0.0) *p* > 0.05 0.0 (0.0–0.0) *p* > 0.05 0.0 (0.0–0.0) *p* > 0.05 0.0 (0.0–0.0) *p* > 0.05 0.0 (0.0–0.0) *p* > 0.05 0.0 (0.0–0.0) *p* > 0.05	1.0 (0.0–7.0) *p* < 0.05 0.0 (0.0–2.0) *p* < 0.05 0.0 (0.0–2.0) *p* < 0.05 0.0 (0.0–1.0) *p* > 0.05 0.0 (0.0–1.0) *p* > 0.05 0.0 (0.0–1.0) *p* > 0.05 0.0 (0.0–2.0) *p* < 0.05	8.0 (4.0–11.0) *p* < 0.001 2.0 (0.0–2.0) *p* < 0.001 1.5 (0.0–2.0) *p* < 0.001 1.0 (0.0–2.0) *p* < 0.001 1.0 (0.0–2.0) *p* < 0.001 2.0 (0.0–2.0) *p* < 0.001 2.0 (0.8–2.0) *p* < 0.001
	**NTG**	**SOAG**	**Controls**
QTV TS S NS NI I TI	6.0 (3.0–12.0) < 0.001 2.0 (0.0–2.0) *p* < 0.05 1.0 (0.0–2.0) >0.05 0.0 (0.0–2.0) >0.05 0.0 (0.0–2.0) >0.05 2.0 (0.0-2.0) < 0.05 2.0 (1.0–2.0) < 0.05	11.5 (6.0–12.0) *p* < 0.001 2.0 (2.0–2.0) *p* < 0.001 2.0 (2.0–2.0) *p* < 0.001 2.0 (0.0–2.0) < 0.01 2.0 (0.0–2.0) < 0.05 2.0 (1.3–2.0) *p* < 0.001 2.0 (1.3–2.0) *p* < 0.001	(0.0–0.0) (0.0–0.0) (0.0–0.0) (0.0–0.0) (0.0–0.0) (0.0–0.0) 0.0 (0.0–0.0)

ROC analysis of qualitative RGCL thickness score is shown in Table 6. QTV showed highest AUC for SOAG, POAG and NTG vs. controls, respectively (>0.9).

**Table 6:** AUC of ROC analysis of qualitative RGCL thickness score for each subgroup (OHT, pre-OAG, NTG, POAG, SOAG vs. controls, respectively): total (QTV, qualitative total value) and sectorial (TS, temporal superior; S, superior; NS, nasal superior; NI, nasal inferior; I, inferior; TI, temporal inferior).

		**TS**	**S**	**NS**	**NI**	**I**	**TI**	**QTV**
OHT-controls	AUC	0.528	0.51	0.538	0.508	0.531	0.519	0.519
Pre-OAG-controls	AUC	0.711	0.683	0.654	0.629	0.616	0.667	0.729
NTG-controls	AUC	0.857	0.747	0.737	0.692	0.832	0.895	0.908
POAG-controls	AUC	0.81	0.832	0.794	0.806	0.826	0.882	0.914
SOAG-controls	AUC	0.932	0.9	0.81	0.799	0.93	0.938	0.93

### Comparison Quantitative vs. Qualitative Analysis

**Figure 2:**
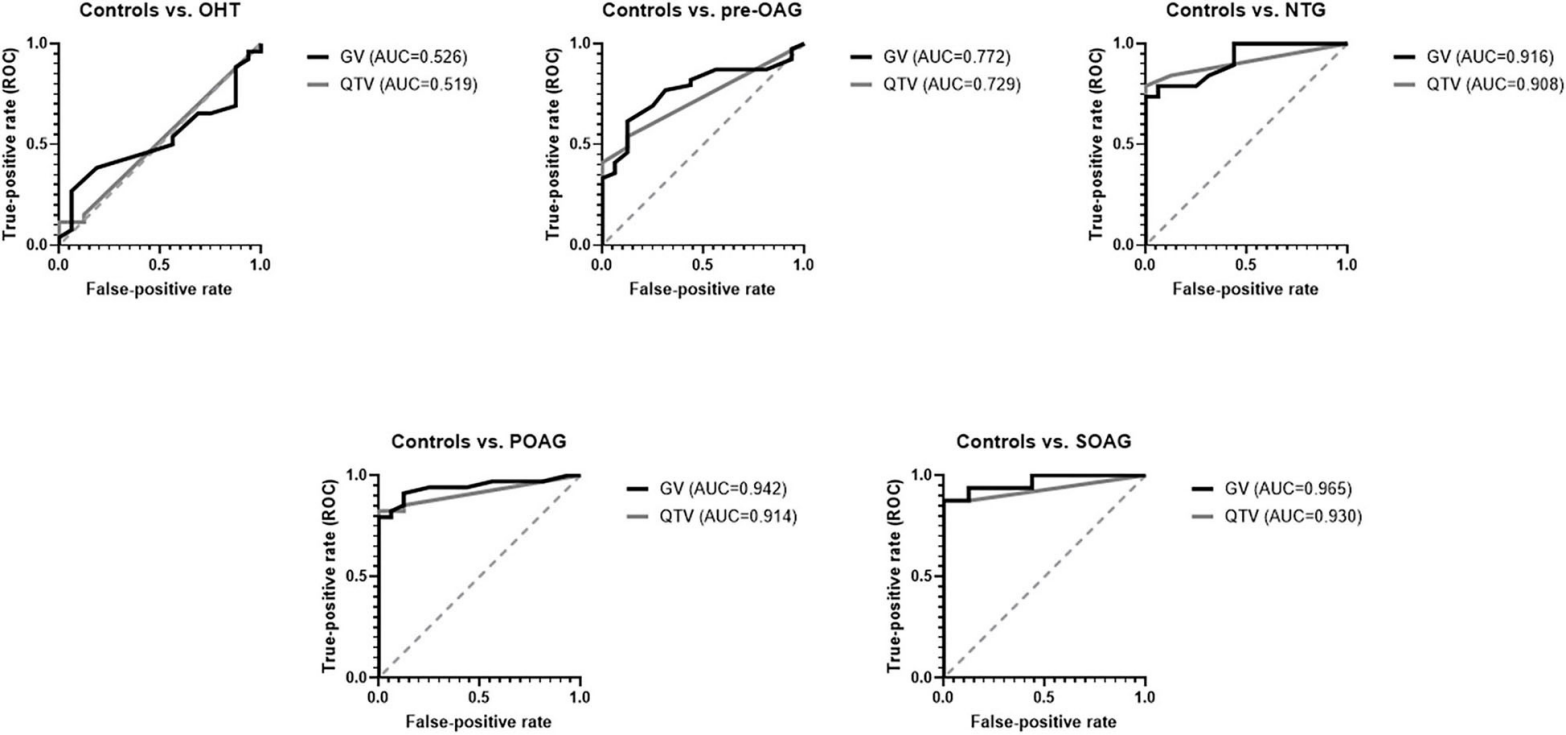
ROC curves for GV and QTV for each subgroup (controls, OHT, Pre-OAG, NTG, POAG, SOAG): GV and QTV yielded similar AUCs.

**Figure 3:**
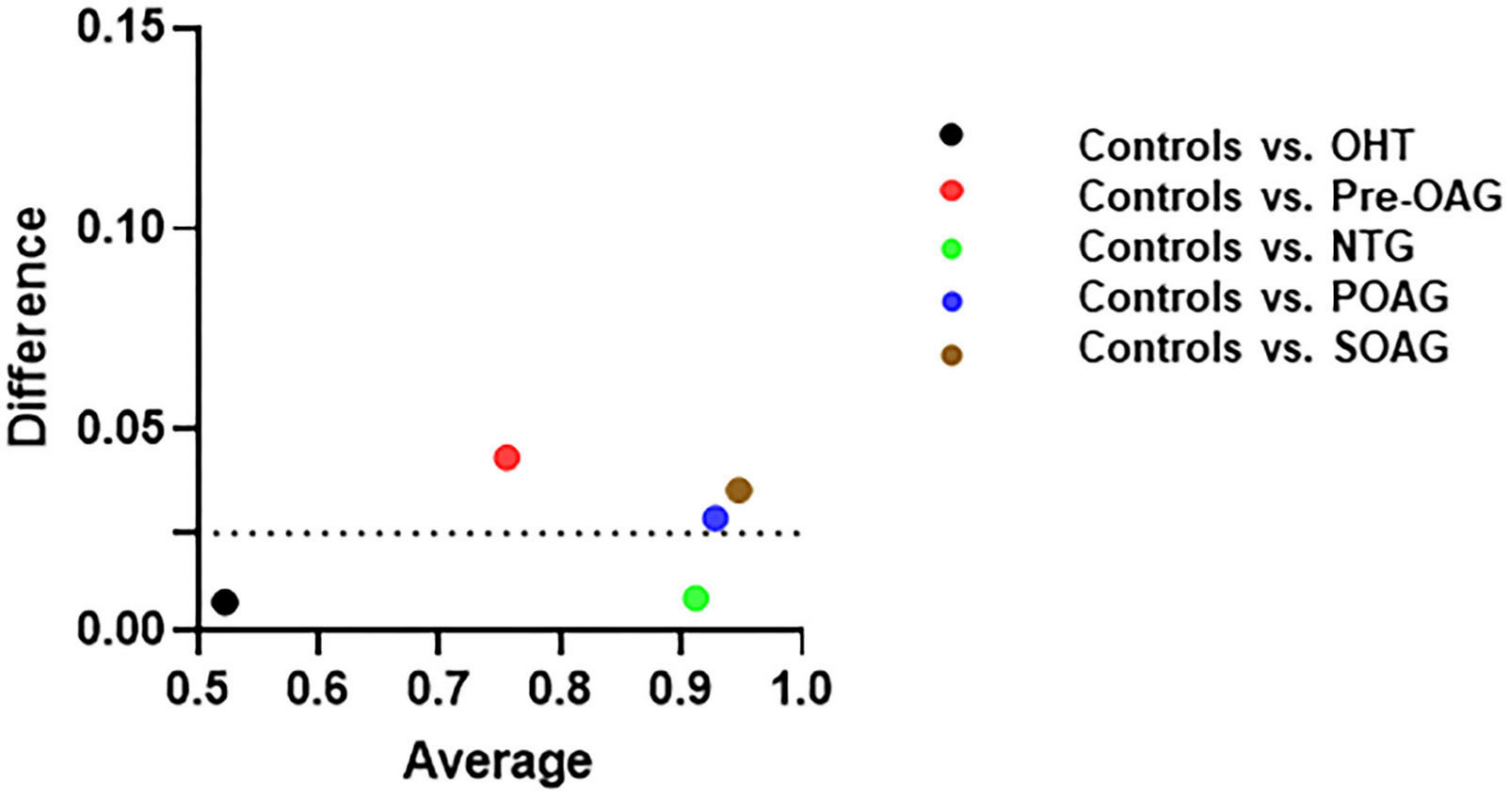
Differences between AUC of quantitative GV (global value) and qualitative. QTV (qualitative total value) plotted against their corresponding average (sum of GV and QTV for each subgroup) in OHT, pre-OAG, NTG, POAG, SOAG vs. controls.

Comparing ROC curves of QTV and GV yielded similar AUC for each subgroup analysis (Figure 2). Mean difference between AUC of GV and QTV (Δ_GV−QTV_) was 0.02 ± 0.016 for the total cohort. Subgroup analysis showed a Δ_GV−QTV_ of 0.007 (OHT vs. control), 0.043 (pre-OAG vs. controls), 0.008 (NTG vs. controls), 0.028 (POAG vs. controls), and 0.035 (SOAG vs. controls). Δ_GV−QTV_ plotted against their corresponding average can be seen in Figure 3 for each subgroup, respectively.

### Qualitative Analysis of Extended Macular Grid

Median and quartiles of extended RGCL thickness score is shown in Table 7. *P*-values for all comparison groups can be seen in Table 7. EQTV differed significantly between pre-OAG, NTG, POAG, SOAG, and controls (*p* < 0.05), respectively. Yet, not significant difference was observed between OHT and controls (*p* > 0.05).

**Table 7:** Extended QTV (qualitative total value) and extended sector scoring of RGCL thickness (median, quartiles) of all subgroups (OHT, pre-OAG, NTG, POAG, SOAG, controls); eQTV, extended qualitative total value; eTS, extended temporal superior; eS, extended superior; eNS, extended nasal superior; eNI, extended nasal inferior; eI, extended inferior; eTI, extended temporal inferior; p-values (p) for comparison of each subgroup with controls (Bonferroni-corrected).

	**OHT**	**Pre-OAG**	**POAG**
eQTV eTS eS eNS eNI eI eTI	5.0 (3.0–12.3) >0.05 1.0 (0.0–3.0) >0.05 1.0 (0.0–3.0) >0.05 1.0 (0.0–2.3) >0.05 0.0 (0.0–3.0) >0.05 0.0 (0.0–1.0) >0.05 0.0 (0.0–3.0) >0.05	20.0 (8.0–42.0) < 0.01 5.0 (1.0–7.0) < 0.01 4.0 (1.0–7.0) < 0.01 4.0 (0.0–7.0) >0.05 2.0 (1.0–7.0) >0.05 3.0 (0.0–7.0) >0.05 4.0 (0.0–7.0) < 0.05	40.0 (31.8–46.3) < 0.001 7.0 (4.8–8.0) < 0.001 7.5 (4.0–8.0) < 0.001 7.0 (4.0–7.0) < 0.001 7.0 (6.0–7.0) < 0.001 7.0 (3.8–8.0) < 0.001 8.0 (6.8–8.0) < 0.001
	**NTG**	**SOAG**	**Controls**
eQTV eTS eS eNS eNI eI eTI	40.0 (32.0–45.0) < 0.001 7.0 (4.8–8.0) < 0.001 7.5 (4.0–8.0) < 0.001 7.0 (4.0–7.0) < 0.05 7.0 (6.0–7.0) < 0.001 7.0 (3.8–8.0) < 0.001 8.0 (6.8–8.0) < 0.001	11.5 (6.0–12.0) < 0.001 2.0 (2.0–2.0) < 0.001 2.0 (2.0–2.0) < 0.001 2.0 (0.0–2.0) < 0.01 2.0 (0.0–2.0) < 0.05 2.0 (1.3–2.0) < 0.001 2.0 (1.3–2.0) < 0.001	43.5 (35.5–47.0) 8.0 (7.0–8.0) 8.5 (7.0–8.0) 7.0 (4.5–8.0) 7.0 (5.3–7.0) 7.0 (5.5–8.0) 8.0 (7.0–8.0)

ROC analysis of extended macular grid is shown in Table 8. EQTV yielded highest AUCs for SOAG, POAG, and NTG vs. controls, respectively (>0.9). Also, sectorial analysis reached high AUC values (Table 8).

**Table 8:** AUC of ROC analysis of extended qualitative RGCL thickness score for each subgroup (OHT, pre-OAG, NTG, POAG, SOAG vs. controls, respectively): total (eQTV, extended qualitative total value) and sectorial (eTS, extended temporal superior; eS, extended superior; eNS, extended nasal superior; eNI, extended nasal inferior; eI, extended inferior; eTI, extended temporal inferior).

		**eTS**	**eS**	**eNS**	**eNI**	**eI**	**eTI**	**eQTV**
OHT-controls	AUC	0.516	0.581	0.594	0.52	0.553	0.519	0.514
Pre-OAG-controls	AUC	0.788	0.765	0.667	0.691	0.685	0.744	0.77
NTG-controls	AUC	0.901	0.929	0.793	0.890	0.908	0.933	0.919
POAG-controls	AUC	0.925	0.910	0.931	0.943	0.938	0.96	0.969
SOAG-controls	AUC	0.949	0.98	0.891	0.924	0.924	0.969	0.973

### Comparison of Quantitative (Macular Grid) Analysis vs. Extended Qualitative Analysis (Using Extended Macular Grid) and Global RNFL

**Figure 4:**
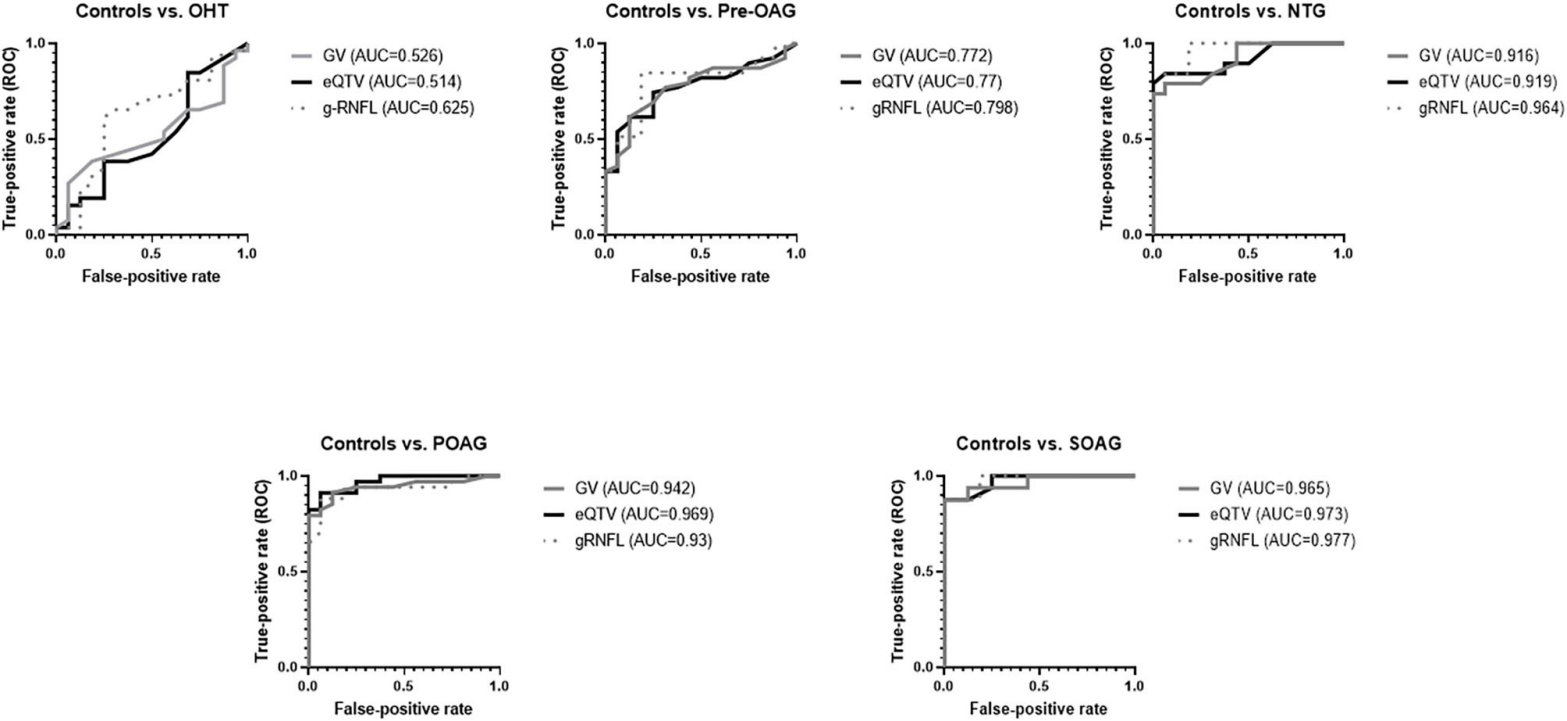
ROC curves for GV, eQTV, and global RNFL (gRNFL) for each subgroup (OHT, Pre-OAG, NTG, POAG, SOAG vs. controls, respectively): In comparison to GV, eQTV yielded higher or similar AUCs.

Comparing ROC curves of GV and eQTV it can be noticed that eQTV yielded higher AUCs than GV in the comparison groups SOAG, POAG, and NTG vs. controls, respectively (Figure 4). At fixed specificity of 100% GV and eQTV had the same sensitivity in SOAG vs. controls; in NTG vs. controls sensitivity of eQTV was even higher (Table 9). In the two remaining subgroups (OHT, pre-OAG), the AUCs of eQTV and GV behaved similarly (Figure 4 and Table 9). In SOAG vs. controls, AUC of global RNFL behaved similarly to AUC of eQTV and GV. Sensitivity and specificity in this subgroup were the same for GV, eQTV, and global RNFL (Table 9). In POAG vs. controls, AUC of global RNFL was smaller than GV and eQTV. In NTG, pre-OAG, and OHT vs. controls, respectively, global RNFL had the highest AUC (Table 9).

**Table 9:** Sensitivity, specificity and AUCs of ROC for GV [μm], QTV, eQTV, and global RNFL [μm] for OHT, pre-OAG, NTG, POAG, and SOAG vs. controls, respectively.

		**Cut-off**	**Sensitivity %**	**Specificity %**	**AUC**
OHT vs. controls	GV	54.5	26.9	93.8	0.526
	QTV	2.5	11.5	100	0.519
	eQTV	2.5	84.6	31.3	0.514
	global RNFL	93.5	61.5	75.0	0.625
Pre-OAG vs. controls	GV	47.5	61.5	87.5	0.772
	QTV	0.5	53.8	87.5	0.729
	eQTV	8.5	74.4	75.0	0.77
	global RNFL	86.5	84.6	81.3	0.798
NTG vs. controls	GV	40.5	73.7	100	0.916
	QTV	2.5	78.9	100	0.908
	eQTV	31	78.9	100	0.919
	global RNFL	86.5	100	81.3	0.964
POAG vs. controls	GV	40.5	79.4	100	0.942
	QTV	2.5	82.4	100	0.914
	eQTV	18	91.2	93.8	0.969
	global RNFL	76.5	88.2	93.8	0.930
SOAG vs. controls	GV	40.5	87.5	100	0.965
	QTV	3.5	87.5	100	0.93
	eQTV	31	87.5	100	0.973
	global RNFL	69.5	87.5	100	0.977

*The cut-off was selected as the point where Youden index had its maximum in each ROC curve (i.e. optimum for sensitivity and specificity)*.

## Discussion

By 2040, the number of patients with glaucoma will have risen to 111.8 million worldwide, visualizing the impact of glaucoma disease (18). Glaucoma is a multifactorial disease with an elevated IOP as most important risk factor, known until now (19). Several studies showed that an increased IOP leads to RGC loss (20–25). One molecular mechanism might be seen in the presence of mechanosensitive Piezo channels within the GCL (26). Piezo channels enable cells to convert a mechanical force into a molecular signaling by detection of e.g., shear stress. After activation of Piezo channels a non-selective influx of cations into the cell generates membrane depolarization and activation of different signaling pathways (Ca^2+^ dependent) (27). Interestingly, the number of retinal Piezo 2 channels within the RGCL was increased after elevating IOP in an animal model (mice) (26). Morphometric measurements of RGCL seem to be prior compared to functional tests (28). It was shown that macular thickness measured by OCT can be considered as a surrogate indicator of RGC loss (29). Especially, in myopic eyes macular GCC thickness measurement performed better than RNFL (30). The present study showed that even looking at the qualitative analysis of RGCL data, yielded a similar diagnostic impact as quantitative data. This has a very practical impact in everyday clinical work where areas of ganglion cell layer thinning can immediately be noticed by an examiner. In addition, implementation of an enlarged macular grid in RGCL analysis was very well-suited to distinguish healthy from glaucoma subjects. It is notable that purely qualitative analysis of the enlarged macular grid—using a point score—yielded higher or at least similar good AUCs compared to the quantitative analysis of conventional smaller macular grid.

Data from earlier studies showed that there is a significant difference in macular thickness volume between healthy subjects and those with glaucoma. However, the diagnostic power did not approach that of the RNFL (31, 32). The diagnostic power was increased by introduction of macular segmentation algorithms, showing similar diagnostic value to RNFL (33, 34). To the best of our knowledge, the present study is the first one, investigating single macular RGCL thickness with the conventional SPECTRALIS® grid and enlarged grid in deviation maps in different types of glaucoma. All recent studies aimed on analysis of the ganglion cell complex (i.e., IPL, GCL, and RNFL) or GCIPL (ganglion cell–inner plexiform layer, i.e., RGCL and IPL) (7, 12, 13). Analyzing average macular GCIPL thickness AUCs of 0.590 (glaucoma suspects vs. controls), 0.668 (early glaucoma vs. controls), and 0.614 (glaucoma suspect and early glaucoma vs. controls) were observed, measured by Cirrus HD-OCT (Carl Zeiss Meditec, Inc., Dublin, CA) (12). AUCs of 0.806 and 0.929 were presented for GCC data, based on measurements with RTVue-100 (Optovue Inc., Fremont, CA) when comparing early glaucoma and advanced glaucoma vs. controls, respectively (35). Next to the differences in measurement of the retinal layers, different grids were used by the two devices: Cirrus HD-OCT measures GCIPL within an elliptical annulus centered on the fovea within an area of 14.13 mm^2^ in six sectors (superotemporal, superior, superonasal, inferonasal, inferior, inferotemporall) and calculates an average value for the whole grid (7, 13). RTVue-100 measures GCC by a square grid of 7 × 7 mm located on the central macula. Quantitative data given by the software include the average thickness and hemifield thickness (superior and inferior) of GCC. Furthermore, the software gives two additional parameters: focal loss volume (FLV) (i.e., average amount of focal GCC loss divided by map area) and global loss volume (GLV) (i.e., sum of all negative fractional deviations within the whole area of the map) (36). Especially, data of the inferior hemifield yielded highest AUCs: AUC 0.75 (pre-OAG vs. controls) (37), AUC of 0.815 (pre-OAG vs. controls) (35), AUC of 0.715 (early glaucoma vs. OHT/controls) (38), and AUC of 0.827 (POAG vs. controls). (38) These results are in accordance with the quantitative data of the macular RGCL thickness in the present study. Highest AUCs were observed in sector TI (AUC = 0.795) and sector I (AUC = 0.78) for controls vs. pre-OAG. In the extended grid sector eTS and eQTV yielded the highest AUCs (0.788, 0.77) for this subgroup. Contrary, the superior hemifield of GCC was observed to be the best for diagnosing glaucoma in eyes with pre-POAG (AUC 0.84 and 0.76) in only one previous study (39).

As an increased IOP induced RGC loss (20–25), devices, investigating thickness of single RGC layer, might improve the diagnostic value of GCC/GCIPL. SPECTRALIS® offers the possibility of analysis of single GCL layer in a defined macular region with different grids. For one special grid an automatic deviation map is generated for each single measurement enabling simultaneously quantitative and qualitative (color-coded) analysis. This macular grid is defined by an elliptic ring consisting of six sectors [superior (S), inferior (I), temporal-superior (TS), temporal-inferior (TI), nasal-superior (NS), and nasal-inferior (NI)]. To best of our knowledge, up to know there is no study available on GCL data of the deviation map and only one study on GCL analysis in eyes with NTG measured by SPECTRALIS® (40). An enhanced stratification of the retina might offer an improved and even finer analysis of pathological alterations. Thus, affections of RGCL might be observed in even earlier stages of disease. Data of the only recent study showed that macular RGCL was significantly thinner in patients with NTG compared to healthy controls (1, 3, 6 mm ETDRS grid). The highest AUC value was reached in superior outer macula sector (0.863), confirming data of the present study (0.9). It is notable that outer sectors yielded generally higher AUC values (0.863, 0.837) than inner sectors (0.747, 0.747) in a previous (1, 3, 6 mm ETDRS grid) (40) and present study [macular grid; eTI (0.933), eQTV (0.919)]. In addition, the outer superior sector of the ETDRS grid yielded the best AUC for differentiating between eyes with primary open-angle glaucoma and controls (AUC = 0.840) (41). Even analysis of macula GCC thickness showed that enlarged grids improved discrimination between glaucoma subjects and controls compared to a smaller standard grid (11). Using the enlarged macular grid, qualitative analysis yielded highest AUCs for sector TI (0.96) and eQTV (0.969; POAG vs. controls) and for sector eS (0.98) and eQTV (0.973, SOAG vs. controls) for the present study cohort. This may reflect the observation that in high-tension open-angle glaucoma loss of ganglion cells are found more to the peripheral macula. In normal-tension glaucoma GCL and visual field defects tend to be more located in the perifoveolar area. This observation may be reflected by our finding that GV an eQTV yielded similar AUC values (0.916 vs. 0.919, respectively).

The study is not without limitations. Patients' cohort is rather small, yet all patients were well-known study participants of the Erlangen Glaucoma Registry and had a follow-up for several years. So, there was no doubt about their clinical classification of disease severity. Furthermore, the extended grid could only be evaluated semi-quantitatively and analysis could not be analyzed in comparison to absolute values. Nevertheless, we used the color-coded information to show, that there is much more information in the whole macular scan than in software's central, standard grid. Color-coded information has the great advantage for clinical examiners to detect deviation of topographic RGCL thickness measurements from normal in a glance. We could show that this clinically comprehensive, qualitative approach showed similar discrimination in the central grid as the absolute, quantitative values. Thirdly, in the analysis the total area of the deviation map was not considered. Probably there would have been even more information on RGCL thickness deviation from normal, in doing so, but we tried to standardize the area of investigation using an extended circle with a fixed prolongation of the software's central, standard grid.

## Conclusion

The results indicate that analysis of RGCL thickness could represent a valuable parameter in glaucoma diagnosis, being comparable to analysis of RNFL. Quantitative analysis showed that AUCs of 0.772 to even 0.965 can be obtained. Analysis of an extended macular region might improve its diagnostic impact. Furthermore, qualitative analysis using the standard macular grid yielded even similar diagnostic impacts compared to quantitative analysis. Clinical practitioners might use this quantitative analysis in their clinical all-day life.

## Data Availability Statement

The original contributions presented in the study are included in the article/supplementary material, further inquiries can be directed to the corresponding authors.

## Ethics Statement

The studies involving human participants were reviewed and approved by the study was approved by the ethics committee of the University of Erlangen-Nürnberg. The patients/participants provided their written informed consent to participate in this study.

## Author Contributions

PL: data analysis and writing of the draft of the manuscript. BH: supervision, revision of the manuscript, and clinical study. RL: clinical study. CM: supervision, revision of the manuscript, and clinical study.

## Conflict of Interest

The authors declare that the research was conducted in the absence of any commercial or financial relationships that could be construed as a potential conflict of interest.
